# Complex breathlessness intervention in idiopathic pulmonary fibrosis (BREEZE-IPF): a feasibility, wait-list design randomised controlled trial

**DOI:** 10.1136/bmjresp-2024-002327

**Published:** 2025-03-22

**Authors:** Michael George Crooks, Caroline Wright, Simon Hart, Victoria Allgar, Anne English, Flavia Swan, Judith Dyson, Gerry Richardson, Maureen Twiddy, Judith Cohen, Andrew Simpson, Chao Huang, Dominic L Sykes, Miriam Johnson

**Affiliations:** 1 Respiratory Research Group, Hull York Medical School, Cottingham, East Yorkshire, UK; 2 Hull University Teaching Hospitals NHS Trust, Hull, UK; 3 Plymouth University, Plymouth, Plymouth, UK; 4 NHS Humber Foundation Trust, Hull, UK; 5 Hull York Medical School, Hull, UK; 6 Centre for Health Economics, University of York, York, UK; 7 University of Hull, Hull, UK

**Keywords:** Interstitial Fibrosis

## Abstract

**Introduction:**

Breathlessness is common and impairs the quality of life of people with idiopathic pulmonary fibrosis (IPF) and non-IPF fibrotic interstitial lung diseases (ILD). We report the findings of a multicentre, fast-track (wait-list), mixed-methods, randomised controlled, feasibility study of a complex breathlessness intervention in breathless IPF and non-IPF fibrotic ILD patients.

**Methods:**

Breathless IPF and non-IPF fibrotic ILD patients were randomised to receive the intervention within 1 week (fast-track) or after 8 weeks (wait-list). The intervention comprised two face-to-face and one telephone appointment during a 3-week period covering breathing control, handheld fan-use, pacing and breathlessness management techniques, and techniques to manage anxiety. Feasibility and clinical outcomes were assessed to inform progression to, and optimal design for, a definitive trial. A qualitative substudy explored barriers and facilitators to trial and intervention delivery.

**Results:**

47 patients (M:F 38:9, mean (SD) age 73.9 (7.2)) were randomised with a recruitment rate of 2.5 participants per month across three sites. The adjusted mean differences (95% CI) for key clinical outcomes at 4 weeks post randomisation were as follows: Chronic Respiratory Questionnaire breathlessness mastery domain (0.45 (−0.07, 0.97)); and numerical rating scales for ‘worst’ (−0.93 (−1.95, 0.10)), ‘best’ (−0.19 (−1.38, 1.00)), ‘distress caused by’ (−1.84 (−3.29, –0.39)) and ‘ability to cope with’ (0.71 (−0.57, 1.99)) breathlessness within the past 24 hours. The qualitative substudy confirmed intervention acceptability and informed feasibility and acceptability of study outcome measures.

**Conclusion:**

A definitive trial of a complex breathlessness intervention in patients with IPF and non-IPF fibrotic ILD is feasible with preliminary data supporting intervention effectiveness.

**Trial registration number:**

ISRCTN13784514.

**WHAT IS ALREADY KNOWN ON THIS TOPIC**

**WHAT THIS STUDY ADDS**

- In this mixed-methods, randomised controlled, feasibility study, we demonstrate that a definitive trial of a complex breathlessness intervention in breathless idiopathic pulmonary fibrosis (IPF) and non-IPF fibrotic interstitial lung disease (ILD) patients is both feasible and acceptable. Additionally, we demonstrate preliminary data supporting the interventions’ effectiveness, warranting confirmation in a phase 3 trial.

**HOW THIS RESEARCH MIGHT AFFECT RESEARCH, PRACTICE OR POLICY**

- This study has confirmed feasibility and informed the design of a multicentre, phase 3, wait-list (fast-track) design randomised controlled trial of the studied complex breathlessness intervention in IPF and non-IPF fibrotic ILD. Our preliminary findings, if confirmed in the definitive trial, suggest that the intervention has the potential to improve breathlessness management for patients living with fibrotic ILD.

## Introduction

 Idiopathic pulmonary fibrosis (IPF) is a progressive and fatal lung disease and other non-IPF fibrotic interstitial lung diseases (ILD) can follow a similar disease course.^1^ Antifibrotic drugs slow the rate of lung function decline, reduce the risk of exacerbations and potentially prolong survival.^2 3^ However, they do not reduce the symptom burden or improve the quality of life of those affected.^4^

Breathlessness is the most common symptom in both IPF and non-IPF fibrotic ILD,^5^ limiting daily activities and adversely affecting quality of life.^6^ Chronic breathlessness profoundly impacts all aspects of an individual’s life and is associated with anxiety, depression^7^ and reduced survival.^8^

Pulmonary rehabilitation (PR) improves symptoms and quality of life for pulmonary fibrosis patients,^9^ but it is not suited or available to all. Palliative care services to support symptom relief are recommended in national guidelines.^10^ However, there is marked variation in service provision for chronic breathlessness and limited evidence to guide the most effective interventions for people with pulmonary fibrosis. Non-pharmacological interventions improve breathlessness mastery and the distress caused by breathlessness due to cardiorespiratory diseases,^11 12^ but people with ILD were under-represented in these trials.^13^

The complex breathlessness intervention in this study has shown promise for improving the severity, ‘distress caused by’ and ‘ability to cope with’ breathlessness, measured using numerical rating scales (NRS).^14^ An adequately powered randomised controlled trial (RCT) in breathless pulmonary fibrosis patients is needed.

To inform the feasibility and optimal design of a definitive trial, we undertook a feasibility study. Based on preliminary data demonstrating that patients benefit from the intervention and following patient and carer feedback, a fast-track (wait-list) design was used.^15^

## Methods

To inform a phase 3 RCT to evaluate the clinical effectiveness and cost-effectiveness of a complex breathlessness intervention in breathless people with IPF and non-IPF fibrotic ILD, we conducted a multicentre, fast-track (wait-list), mixed-methods, feasibility RCT. The study protocol has been published elsewhere^16^ and our methods are summarised here.

### Study population

We recruited patients with chronic breathlessness due to IPF or non-IPF fibrotic ILD over 18 months to provide sufficient data to assess feasibility parameters. Eligible participants provided written informed consent and were randomised 1:1, stratified by site, to either fast-track or wait-list groups using random permuted blocks. It was impossible to blind patients, clinicians or trial staff to treatment group allocation.

Participants were recruited from three centres in England (one tertiary ILD service, one urban district hospital and one rural district hospital) between July 2018 and January 2020. Eligible participants were aged ≥50 years with a multidisciplinary team diagnosis of IPF or other fibrotic ILD, had mMRC breathlessness grade 3 or 4 and had oxygen saturations ≥90% while breathing air or their usual oxygen prescription. Those with another cardiorespiratory disease as the primary cause for breathlessness and those having completed PR or breathlessness clinic attendance within 3 months of screening were excluded.

Initially, only those with IPF were eligible. Due to data demonstrating the parallels between IPF and other fibrotic ILD and the potential for palliative interventions to improve breathlessness in this population,^1 17^ eligibility criteria were amended in March 2019 to include people with non-IPF fibrotic ILD (fibrotic non-specific interstitial pneumonia, fibrotic hypersensitivity pneumonitis, fibrotic organising pneumonia and unclassifiable fibrotic ILD).

### Study intervention

The intervention was delivered during three consultations with a trained practitioner (Any healthcare professional who was trained in delivery of the breathlessness intervention was able to deliver the intervention. In this feasibility study, this role was undertaken by allied health professionals including physiotherapists and an occupational therapist.). The first two were face-to-face, lasted approximately 1 hour, and took place 1 week apart. A final telephone consultation lasting 10–15 min was conducted 1 week later. The intervention was individually tailored, but included the following core aspects:

- Breathing control techniques (eg, diaphragmatic and rectangular breathing).^18^,^21^
- A handheld fan with instructions for use.^21 22^
- Pacing and breathlessness management during everyday activities, including positions for recovery from exertional breathlessness and information on the importance of exercise.^18 20 23^
- Techniques to promote relaxation and manage anxiety and panic (eg, preparation and positions to relax, and mindfulness).^24^

All participants were given a breathlessness information leaflet (adapted, with permission, from the Breathlessness Intervention Service at Addenbrooke’s Hospital, Cambridge).

Delivery of the breathlessness intervention core components and breathlessness leaflet provision was recorded to monitor fidelity.

### Outcomes

#### Feasibility

Feasibility outcomes related to:

- Recruitment (eligibility to consent ratio; recruitment rate; and retention/follow-up rates at 4, 8, 12 and 16 weeks).
- Data quality (completion of questionnaires and other assessments at baseline, 4, 8, 12 and 16 weeks and patterns of missing data).
- Intervention (adherence in delivery/uptake and acceptability to participants).

Anonymous, paper ‘Invitation’ and ‘Study Experience’ Surveys were distributed during the first and last visits, respectively, to capture participants’ views about the recruitment process and overall experience of participation.

#### Effectiveness

The following clinical outcomes were measured for a signal of effectiveness and to inform outcome selection and sample size for a phase 3 trial:

##### Breathlessness

The breathlessness mastery domain of the Chronic Respiratory Disease Questionnaire (CRQ).^25^ The mastery domain has been used successfully as a primary outcome in clinical trials and patient and public feedback reported completion of the full CRQ to be burdensome to participants with this level of breathlessness.^19^ Consequently, only the mastery domain was measured.

NRS (score 0–10) for ‘best breathlessness’, ‘worst breathlessness’, ‘distress caused by breathlessness’ and ‘coping with breathlessness’ are all within the past 24 hours.^1826^,^28^ NRS scores are unidimensional scales recommended for breathlessness measurement in advanced disease,^27^ with established minimal clinically important differences.^29^

##### Quality of life

St Georges Respiratory Questionnaire for patients with IPF (SGRQ-I),^30^ EQ-5D-5L and EQ-VAS^31^ were used to measure disease specific and generic health-related quality of life.

##### Mood

The hospital anxiety and depression scale (HADS)^32^ was used to measure anxiety and depression.

##### Physical activity, functional status and exercise capacity

The Australian-modified Karnofsky Performance Status (AKPS)^33^ was used to assess functional status and exercise capacity was assessed using the incremental shuttle walk test (ISWT).^34^

Physical activity was measured during normal daily life using an ankle-worn Actigraph GT3XP-BT (Actigraph, Florida, USA) for 7 days. Minimum wear time of ≥10 hours on any given day was required for the data inclusion. The number of valid days, wear time, step count, sedentary time, light activity time and moderate/vigorous activity time were reported descriptively according to Freedson *et al*.^35^

### Pulmonary function

Pulmonary function tests were conducted in accordance with American Thoracic Society/European Respiratory Society Guidelines.^36^

### Health economics

Health service utilisation was recorded including the following: GP attendance, practice nurse attendance, out-patient appointment attendance (consultant), specialist nurse review (out-patient or home-visit), emergency department attendance, hospital admission, hospice admission. The EQ-5D-5L and EQ-VAS and ICECAP supportive care measure (ICECAP-SCM) were collected to assess the feasibility of QALY calculation.

### Qualitative substudy

In-depth, semistructured interviews were conducted with a purposive sample of patients and carers by two post-doctoral researchers (no previous knowledge of the participants) to determine the acceptability of trial processes and the intervention. Interviews were also conducted with recruiters and practitioners at each site to investigate their experience of trial processes and the intervention. A theoretical framework was used^37^ to explore determinants of intervention concordance.

### Data analysis

#### Feasibility randomised controlled trial

The trial is reported in accordance with the CONSORT 2010 statement extension to pilot and feasibility trials.^38^ Analyses were conducted on an intention-to-treat basis.

Participant characteristics are summarised descriptively. Mean and 95% CIs are reported for key feasibility and clinical outcomes. Candidate definitive trial primary outcomes (CRQ mastery and NRS breathlessness scores) are plotted graphically as change from baseline by the treatment group at each time point. The corresponding effect sizes are calculated with 95% CIs at the primary end point (visit 1) to inform the definitive trial sample size.

Descriptive statistics are reported for other clinical outcomes. Due to this being a feasibility study, no formal statistical tests were undertaken.

#### Qualitative substudy

Interview data were transcribed, anonymised and subject to reflexive thematic analysis^39^ to identify and interpret themes in accordance with the aim (acceptability of trial processes and the intervention). Analysis was undertaken by two researchers with input from a third over a period of discussion to inductively generate, review and refine analytic themes through the lens of our theory. NVivo (V.11) software was used for analysis (QSR International 2012). Patient/carer dyad transcripts were analysed together to capture content and interactions.

### Patient and public involvement

Pulmonary Fibrosis Support Group members contributed to study inception and design, including informing the research question and wait-list design. The research team included a patient representative who contributed to the drafting of study documents. A patient attended trial management group meetings and study progress was discussed at quarterly Pulmonary Fibrosis Support Group meetings.

### Adverse events

Adverse events are defined in the full study protocol^16^ and were reported in accordance with UK NHS Research Ethics Service Guidelines.

## Results

### Multicentre, fast-track (wait-list), randomised controlled feasibility trial

#### Study recruitment

Three sites opened to recruitment between July 2018 and February 2020 and 96 potential participants were assessed for eligibility with 64 considered eligible (eligibility rate 66.7%; 95% CI 56.3% to 76%). 50 participants consented (consent rate 78.1%; 95% CI 66.0% to 87.5%) and 47 patients were randomised (25 in wait-list group and 22 in fast-track group). The recruitment rate across study sites is presented in table 1.

**Table 1:** Study recruitment across study sites

Site description	Number of recruits(n)	Duration site open for recruitment (months)	Recruitment rate(n per month)
Tertiary ILD Centre	44	20	2.2
Urban District General Hospital	4	5	0.8
Rural District General Hospital	2	17	0.12

**Figure 1:**
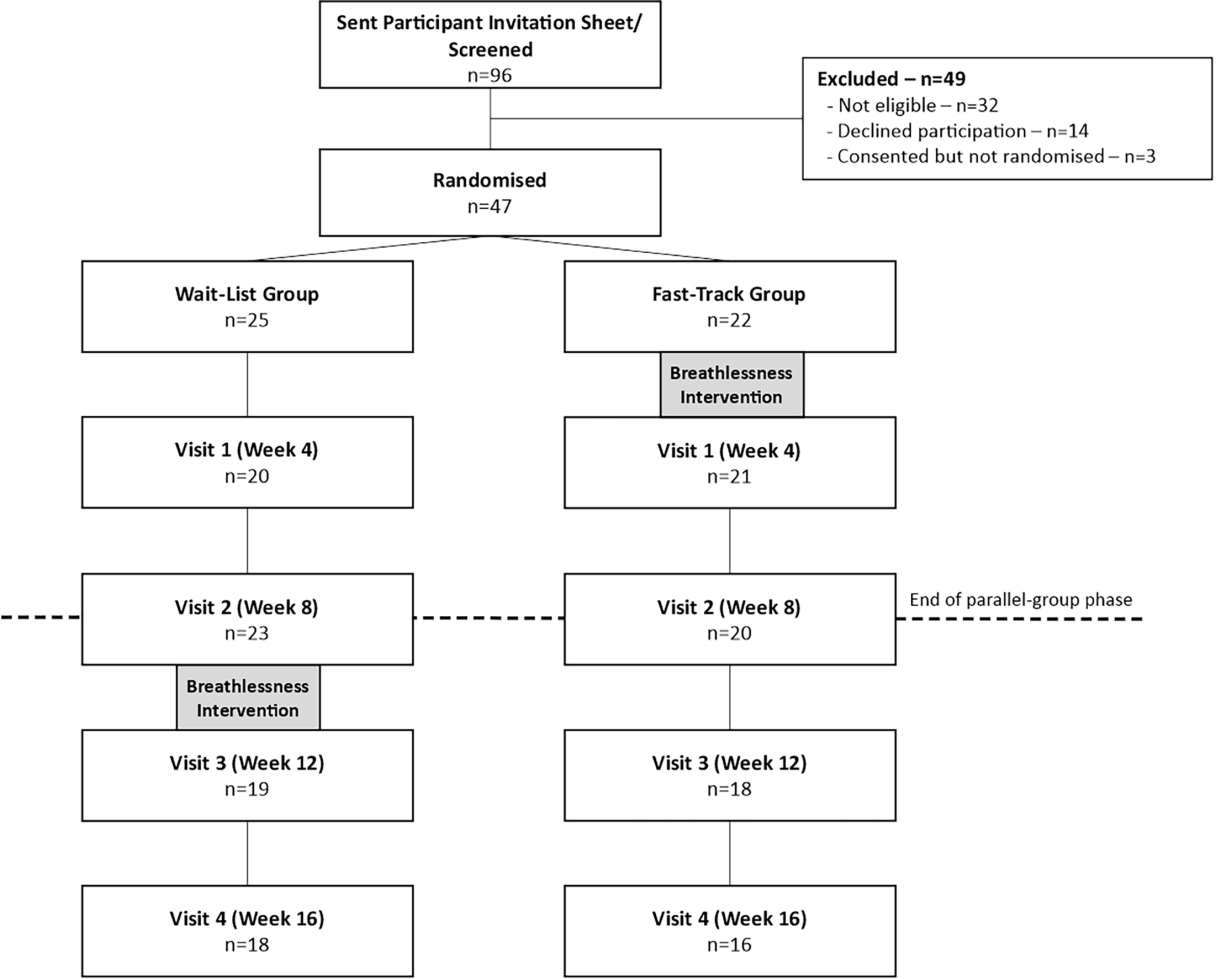
Consort diagram showing participant recruitment and retention throughout the trial.

Retention at 4 weeks (proposed definitive trial primary outcome time point) was 87% (41/47). Figure 1 shows participant flow.

#### Participant characteristics

Randomised participants (n=47) had a mean age of 73.9 (SD 7.2) years and 80.8% (n=38) were male. 74.5% (n=35) of participants had IPF and 19.1% (n=9) had non-IPF fibrotic ILD. A diagnosis was unavailable for three participants but investigators confirmed fibrotic ILD. Participant characteristics and baseline assessments are summarised in table 2. 11 (23%) participants had previously participated in pulmonary rehabilitation (5/22 participants in the fast-track arm and 6/25 in the wait-list arm).

**Table 2:** Participant characteristics

	Wait-list (n=25)	Fast-track (n=22)	Overall (n=47)
Age—mean (SD)			
Age in years	72.5 (7.2)	75.6 (6.8)	73.9 (7.2)
Gender—n (%)			
Male	21 (84.0%)	17 (77.3%)	38 (80.9%)
Female	4 (16.0%)	5 (22.7%)	9 (19.1%)
Ethnicity—n (%)			
White British	25 (100%)	22 (100%)	47 (100%)
Other	0 (0%)	0 (0%)	0 (0%)
Smoking history—n (%)			
Non-smoker	4 (16.0%)	6 (27.3%)	10 (21.3%)
Past smoker	20 (80.0%)	16 (72.7%)	36 (76.6%)
Current smoker	1 (4.0%)	0 (0%)	1 (2.1%)
Pack years—mean (SD)	27.3 (28.7)	41.1 (21.4)	33.3 (26.4)
Diagnosis—n (%)			
IPF	19 (76.0%)	16 (72.7%)	35 (74.5%)
Other fibrotic ILD	4 (16.0%)	5 (22.7%)	9 (19.1%)
Missing data	2 (8.0%)	1 (4.5%)	3 (6.4%)
BMI—n	n=25	n=21	n=46
BMI—mean (SD)	27.6 (3.9)	27.8 (4.0)	27.7 (3.9)
mMRC—n (%)			
3	18 (72.0%)	13 (59.1%)	31 (66.0%)
4	7 (28.0%)	9 (40.9%)	16 (34.0%)
Spirometry data—mean (SD)	n=22	n=19	n=41
FVC	2.31 (0.65)	2.41 (0.65)	2.37 (0.64)
FVC % predicted	63.5 (19.1)	76.1 (17.8)	70.2 (19.0)

#### Feasibility outcomes

Feasibility outcomes were assessed against predefined, stop-go criteria. All feasibility criteria were met, indicating that a definitive trial is feasible.

The eligibility:consent ratio was 1.28:1 and the recruitment rate was 2.5 patients per month. Participant retention at visit 1 (week 4) was 87% and 72% at visit 4. Intervention adherence was 94% and 100% for the two face-to-face breathlessness clinic attendances and 94% for the telephone appointment. Feasibility outcomes and their respective stop-go criteria are presented in online supplemental table 1.

#### Key clinical outcomes (candidate primary outcomes for a definitive trial)

**Figure 2:**
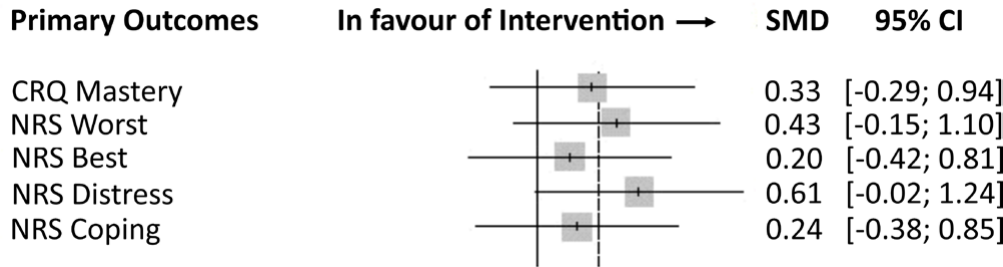
Forest plot of calculated standardised effect sizes for definitive trial candidate primary outcomes.

**Figure 3:**
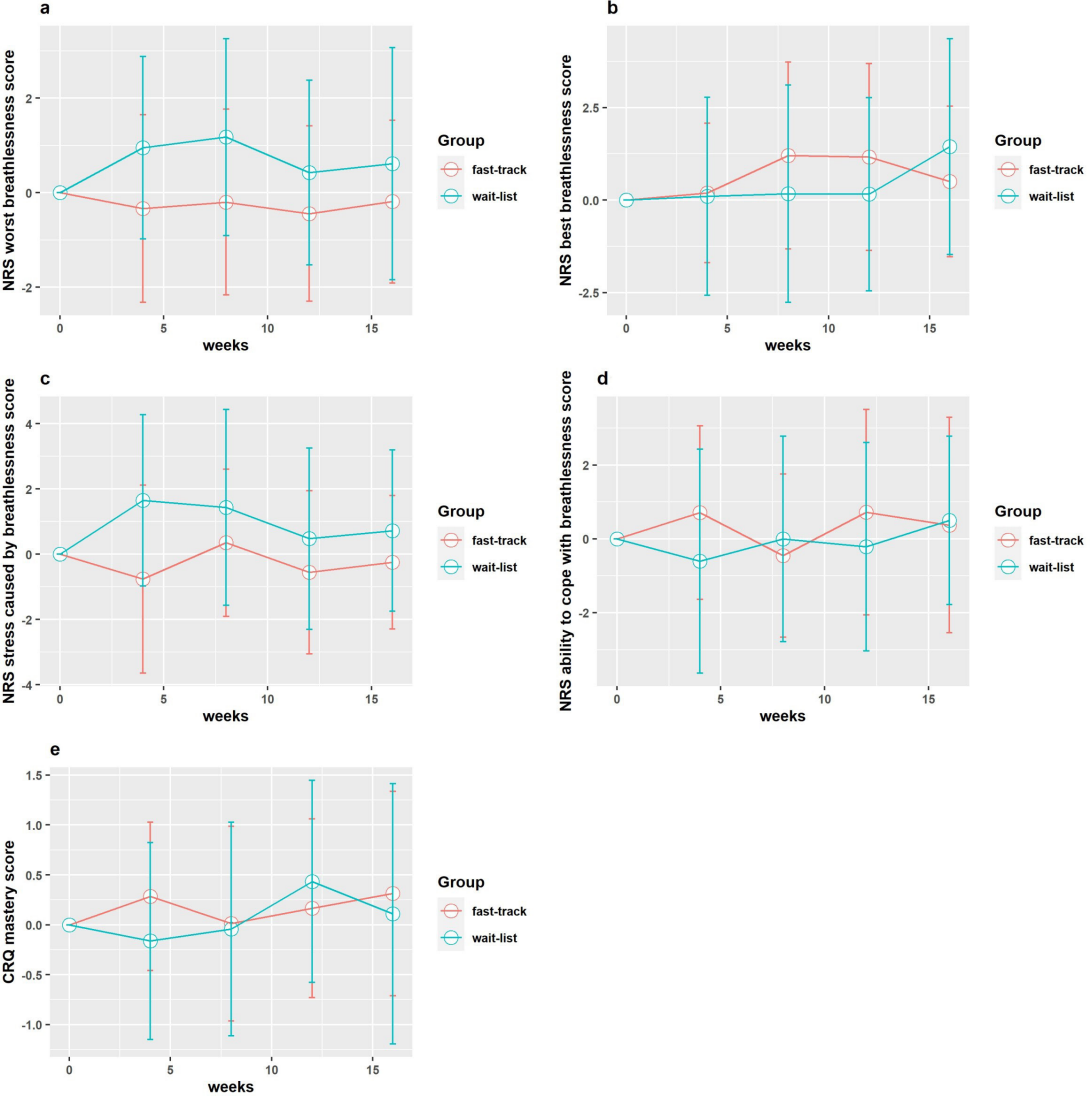
Change from baseline at subsequent study visits for the definitive trial candidate primary outcome measures. (a) NRS worst breathlessness score: changes from baseline (positive changes indicate getting worse) (b) NRS best breathlessness score: changes from baseline (positive changes indicate getting worse) (c) NRS distress caused by breathlessness score: changes from baseline (positive changes indicate getting worse). (**d**) NRS ability to cope with breathlessness score: changes from baseline (positive changes indicate getting better) (e) CRQ mastery score: changes from baseline (positive changes indicate getting better).

The key clinical outcomes and candidate primary outcomes for a definitive trial were the CRQ mastery domain score and NRS scores for ‘worst’, ‘best’, ‘distress caused by’ and ‘ability to cope with’ breathlessness during the past 24 hours. The unadjusted and adjusted between-group differences at visit 1 and the calculated effect sizes are presented in table 3 and figure 2. The change from baseline for key clinical outcomes at each study time point is presented in figure 3.

**Table 3:** The unadjusted and adjusted between-group differences of the proposed primary outcomes at visit 1, with CIs and calculated effect size

Proposed primary outcome	Unadjusted mean difference (95% CI) at visit 1	Mean difference(95% CI) at visit 1 adjusting for baseline score	Calculated effect size
CRQ: breathlessness mastery domain	0.45 (−0.32, 1.23)	0.45 (−0.07, 0.97)	0.38
NRS breathlessness score: worst	−0.80 (−1.83, 0.24)	−0.93 (−1.95, 0.10)	0.47–0.55
NRS breathlessness score: best	−0.35 (−1.62, 0.92)	−0.19 (−1.38, 1.00)	0.09–0.16
NRS breathlessness score: caused distress	−1.55 (−3.05, –0.05)	−1.84 (−3.29, –0.39)	0.70–0.84
NRS breathlessness score: how well coped	0.53 (−0.77, 1.82)	0.71 (−0.57, 1.99)	0.21–0.28

#### Other clinical outcomes

A large proportion of missing data was observed for the ISWT, lung function tests and activity monitoring data. This was attributed to reduced acceptability and feasibility of performing these assessments in participants with chronic breathlessness and limited performance status.

There was no evidence of disease progression based on forced vital capacity measurement at baseline, week 8 and week 16. There were numerical decreases in the mean HADS scores from baseline in both depression and anxiety domains following intervention delivery in both fast-track and wait-list groups. Other clinical outcome data are presented in online supplemental tables 2–7.

#### Safety

There were five serious adverse events (SAE) reported during the study (wait-list=2, fast-track=3). None were related to the intervention or study procedures. Three participants died (wait-list=1, fast-track=2). Adverse event data can be seen in online supplemental table 8a,b.

#### Health economic feasibility

Baseline EQ-5D-5L questionnaires were completed for all participants. Follow-up response rates were lower but acceptable (16-week follow-up response rate: 73% for fast-track group and 72% for wait-list group). EQ-5D-5L and EQ-VAS scores remained relatively unchanged between baseline and visit 1 in the fast-track group but increased numerically between visits 2 and 3 in the wait-list group (online supplemental table 9).

Resource use questionnaires had high response rates across all visits. Healthcare resource utilisation data are presented in online supplemental tables 10–13. ICECAP-SCM data are presented in online supplemental table 14.

### Qualitative substudy

16 participants were interviewed: 6 patients (5 males, age range 62–80 years, IPF duration 2–6 years), 3 carers (all female) and six clinical practitioners (three research nurses and three physiotherapists). All carers were interviewed with patient-participants.

**Figure 4:**
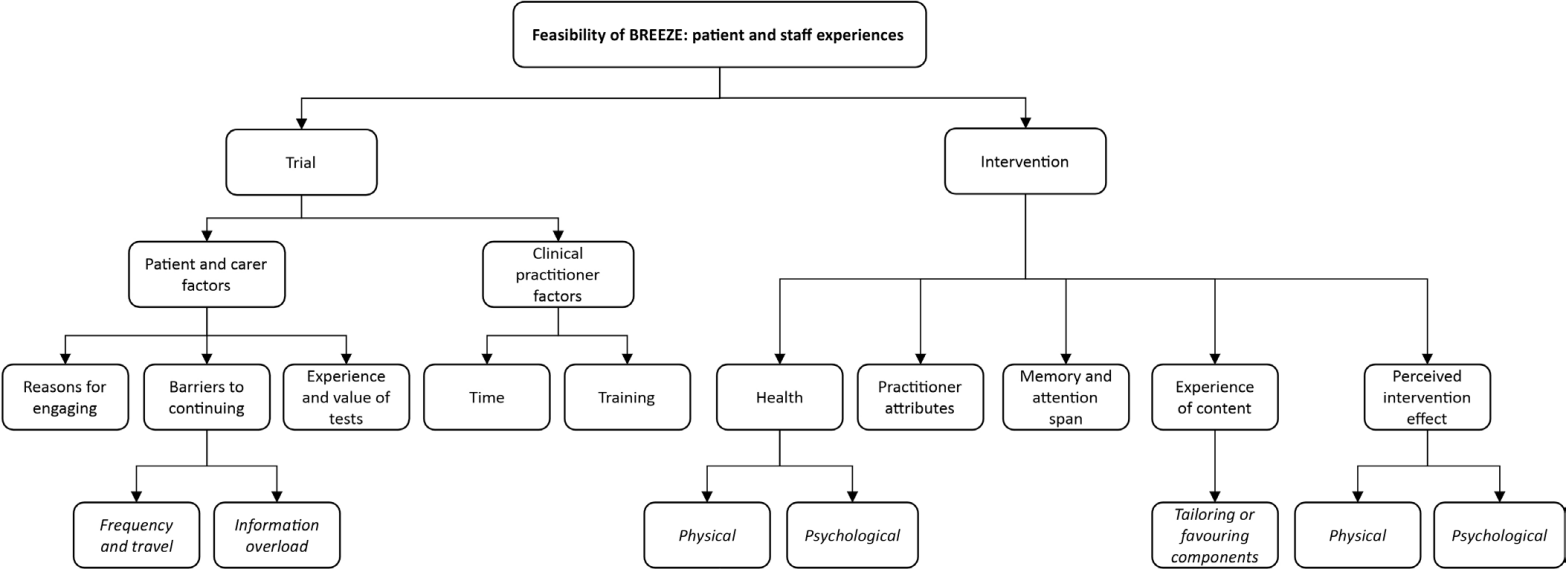
Qualitative substudy themes and subthemes.

Data were themed around (1) acceptability of trial processes (study participation and outcome measures) and (2) acceptability of the intervention. Figure 4 illustrates themes and subthemes which are described below.

#### Trial

Patients and carers had positive experiences of recruitment and reported **reasons *for engaging***, such as self-help and the possibility of helping others in the future. The frequency of and travel to appointments and feeling overwhelmed by provided information (‘information overload’) were ***barriers to continuing*** with the trial. Travel and appointment frequency were alleviated by the offer of home visits. The large amount of information offered, referred to by one patient as ‘a bit of a pain….’ was mitigated by summarising and tailoring the information for the individual. ***Experience of the tests*** (outcome assessments) was generally positive with participants understanding the need and relevance. The main negative experience was the ISWT.

Practitioners were similarly positive about trial procedure, other than (1) the ***time*** associated with increased workload (“the only challenge … trying to fit them in within the week”) and (2) the need for repeated trial and intervention ***training*** when new staff started.

Patients’, carers’ and practitioners’ experience of the intervention was overwhelmingly positive but the greatest barrier to engagement was ***physical and psychological health***. A practitioner reported, “if people are more poorly, they’re probably, not, not engaged as well as other[s]”, and a patient shared, “you’re in a depression, you don’t think about anything, you don’t think logically at all … you can’t manage that [the condition] because it manages you.” Patients and carers cited ***practitioner attributes*** as facilitators to intervention engagement, including enthusiasm, flexibility, knowledge and availability (one patient said, “*caring … we really rated him*… [he was] *just at the end of the phone*”). Patients reported poor ***memory and attention span*** as a barrier to engaging with the intervention. In relation to breathing exercises, one participant reported, “they said think of a window and then … I’d forgotten … too much to absorb”. Participants described having favourite elements of the intervention, for example, the relaxation CD was referred to by one patient as “absolutely superb”, whereas another said the handheld fan was “the most constructive…”. This linked with ***tailoring the intervention*** to an individual’s lifestyle and physical needs/abilities (eg, timing and duration of physical activity). Finally, the ***physical and psychological impact of the intervention*** was experienced as beneficial by most participants. Reported benefits included greater control over physical symptoms which had an impact on mood. A carer said, “it’s been a great boost”, a patient noticed he was “better in myself” and a practitioner reported, “he was so elated … because he had a bit of control over it”.

Study invitation and experience survey data indicated that participants understood the purpose of the trial and the breathlessness management techniques. Most participants described their overall experience of taking part in the study as good (23/25, 92%); two described their experience as fairly good. The main challenges associated with the trial were travel, number and, for many, the arduous nature of appointments, the number of questionnaires and difficulties with those assessments requiring physical exertion such as the ISWT.

A summary of the mixed-methods analysis is detailed in online supplemental table 15.

## Discussion

A definitive randomised controlled trial to assess the effectiveness and cost-effectiveness of a holistic complex breathlessness intervention in patients with pulmonary fibrosis is feasible and acceptable to patients and healthcare practitioners. The trialled breathlessness intervention led to numerical improvements in all key clinical outcomes, providing a signal of effectiveness that warrants definitive assessment in a phase 3 trial.

In this feasibility study, we achieved predefined stop-go criteria for all outcomes except recruitment rate and participant retention to 16 weeks, both of which achieved intermediate ratings. All data quality outcomes met their prespecified targets and the intervention was found to be both feasible to deliver with fidelity and acceptable to patients.

Recruitment was notably higher in the tertiary ILD centre than in district hospitals and it was observed that the pulmonary fibrosis patients in district hospital sites are often referred to the tertiary centres for diagnosis and treatment. To address the intermediate outcome for recruitment and retention feasibility criteria, the definitive trial should ensure that enough tertiary ILD centres are included as sites to meet recruitment targets.

In terms of demonstrating the signal of intervention effectiveness, candidate primary outcome measures all demonstrated numerical improvement with a moderate and fair effect size for CRQ breathlessness mastery and NRS ‘worst breathlessness’ and equivalent to a moderate minimum clinically important difference (MCID).^29^ Participants reported finding the CRQ burdensome, even though only the mastery domain was used. The improvement for the ‘distress caused by breathlessness’ NRS represented a moderate effect size but this dimension has not previously been validated in this population. Therefore, we suggest that the optimal primary outcome for the definitive trial is NRS ‘worst breathlessness’.^27 28^ Based on the findings of this study, to demonstrate the MCID (1-point change),^27 29^ a minimum sample size of 62 participants per group completing the trial would be required for 90% power and 0.05 significance in the definitive trial. The recruitment target for the definitive trial will need to take account of the retention rate observed in this feasibility study to achieve the required number of completing participants.

The missing data for effort-dependent assessments such as ISWT appear surprising at first glance given the reasonable completion rates in clinical practice during pulmonary rehabilitation. However, our study participants are those with severe breathlessness and poor performance status, a group that often do not attend or complete pulmonary rehabilitation. These missing data reflect the lack of acceptability of this assessment to our study participants.

The qualitative substudy and mixed-methods analysis identified both barriers and facilitators to definitive trial recruitment. Simplifying study visits by reducing questionnaire burden, remote data collection and discontinuing effort-dependent assessments were considered important.

Our data provide positive signs of intervention effectiveness that warrant confirmation in a definitive phase 3 trial. Our findings inform the design of such a trial involving patients with chronic breathlessness caused by IPF or non-IPF fibrotic ILD.

## Conclusions

A definitive trial is feasible and acceptable to patients and healthcare practitioners with some minor adaptations to trial design. A primary outcome of NRS (worst in the past 24 hours) has been identified and with a signal of benefit. These data will inform the design and delivery of a definitive RCT of the complex breathlessness intervention in patients with pulmonary fibrosis.

## supplementary material

10.1136/bmjresp-2024-002327 online supplemental file 1

## Data Availability

All data relevant to the study are included in the article or uploaded as supplementary information.
