# Supplementary material for: Complex breathlessness intervention in idiopathic pulmonary fibrosis (BREEZE-IPF): a feasibility, wait-list design randomised controlled trial
Source: BMJ Open Respir Res. 2025 Mar 22;12(1):e002327. doi: 10.1136/bmjresp-2024-002327 (PMC11931949; doi:10.1136/bmjresp-2024-002327)
Supplement: online supplemental file 1 [file bmjresp-12-1-s001.pdf]

Supplementary Materials

A feasibility, wait-list design randomised controlled trial of a complex breathlessness intervention in idiopathic pulmonary fibrosis (BREEZE-IPF)

Authors

Michael G Crooks[1], Caroline Wright[1], Simon P Hart[1], Victoria Allgar[2], Anne English[3], Flavia Swan[4], Judith Dyson[4], Gerry Richardson[5], Maureen Twiddy[8], Judith Cohen[7], Andrew Simpson[6], Chao Huang[7], Dominic L Sykes[1], Miriam J Johnson[4]

1. Respiratory Research Group, Hull York Medical School
2. University of York
3. NHS Humber Foundation Trust
4. Wolfson Palliative Care Research Centre, Hull York Medical School, University of Hull
5. Centre for Health Economics, University of York
6. School of Sport, Exercise and Rehabilitation Sciences, University of Hull
7. Hull Health Trials Unit, University of Hull
8. Institute of Clinical and Applied Health Research, Hull York Medical School, University of Hull

Contents

Supplementary Table 1. Results of Feasibility outcomes 3

Supplementary Table 2. Spirometry data measured at baseline, visit 2 and visit 4. 4

Supplementary Table 3. The hospital anxiety and depression scale (**HADS**) measured at baseline, visit 1, 2, 3 and 4. 5

Supplementary Table 4. AKPS measured at baseline, visit 1, 2, 3 and 4. 5

Supplementary Table 5. Incremental Shuttle Walk Test (ISWT) measured at baseline, visit 1, 2, 3 and 4. 6

Supplementary Table 6. Physical activity data derived from the Actigraph GT3XP-BT device at baseline and visits 1,2,3, and 4. Physical activity intensity was defined as per Freedson et al. (1998)[1]. 7

Supplementary Table 7. SGRQ-I measured at baseline, visit 1, 2, 3 and 4. 9

Supplementary Tables 8a and 8b. Adverse event data for both groups. 10

Supplementary Table 9. EQ-5D-5L measured at baseline, visit 1, 2, 3 and 4. 11

Supplementary Table 10. Health Care Utilisation measured at **Visit 1** 12

Supplementary Table 11. Health Care Utilisation measured at **Visit 2** 13

Supplementary Table 12. Health Care Utilisation measured at **Visit 3** 14

Supplementary Table 13. Health Care Utilisation measured at **Visit 4** 15

Supplementary Table 14. ICECAP-SCM data for fast-track and wait-list groups at all study visit. V1: Visit 1; V2: Visit 2; V3: Visit 3; V4: Visit 4. 16

Supplementary Table 15. Mixed Methods Analysis........ 17

References 19

Supplementary Table 1. Results of Feasibility outcomes

**Recruitment**	**Target**	**Result**
Eligibility:Consent Ratio	Red >8:1	
	Amber 8:1-3:1	1.28:1
	Green <3:1	
Recruitment rate - participants per month	Red <2	
	Amber = 2	2.5
	Green >3	
Participant retention – 4 weeks	Red <70%	
	Amber 70-80%	87%
	Green >80%	
Participant retention – 16 weeks	Red <70%	
	Amber 70-80%	72%
	Green >80%	
**Data Quality – Proposed Primary Outcome**		
Completion rate of CRQ – Baseline	Red <85%	
	Amber 85-95%	100%
	Green >95%	
Completion Rate of breathlessness NRS score - Baseline	Red <85%	
	Amber 85-95%	100%
	Green >95%	
Completion Rate of CRQ – 4 weeks	Red <75%	
	Amber 75-85%	87%
	Green >85%	
Completion Rate of breathlessness NRS score – 4 weeks	Red <75%	
	Amber 75-85%	87%
	Green >85%	
**Data Quality – Other Patient Outcomes**		
Completion rate of other patient outcomes - Baseline	Red <75%	
	Amber 75-85%	100%
	Green >85%	
Completion rate of other patient outcomes – 4 weeks	Red <70%	
	Amber 70-80%	87%
	Green >80%	
**Intervention Adherence**		
Visit 1	Red <80%	94%
Visit 2	Amber 80-90%	100%
Visit 3	Green >90%	94%
**Intervention Fidelity** – Breathing Control		
Visit 1	Red <80%	82%
Visit 2	Amber 80-90%	81%
Visit 3	Green >90%	79%
**Intervention Fidelity** – Pacing		
Visit 1	Red <80%	100%
Visit 2	Amber 80-90%	100%
Visit 3	Green >90%	100%
**Intervention Fidelity** – Fan Therapy		
Visit 1	Red <80%	100%
Visit 2	Amber 80-90%	100%
Visit 3	Green >90%	97%
**Intervention Fidelity** – Anxiety/ Relaxation Techniques		
Visit 1	Red <80%	97%
Visit 2	Amber 80-90%	84%
Visit 3	Green >90%	83%
**Intervention Fidelity** – Exercise Advice		
Visit 1	Red <80%	100%
Visit 2	Amber 80-90%	94%
Visit 3	Green >90%	93%

Supplementary Table 2. Spirometry data measured at baseline, visit 2 and visit 4.

		Wait list – mean (SD)	Fast track - mean (SD)
**Baseline**	*Completed cases*	*n=22*	*n=19*
	FEV1	1.85 (0.49)	1.90 (0.53)
	FEV1 predicted	2.62 (0.58)	2.50 (0.66)
	FEV1 % predicted	63.7 (22.6)	77.3 (17.0)
	FVC	2.31 (0.65)	2.41 (0.65)
	FVC predicted	3.42 (0.77)	3.15 (0.80)
	FVC % predicted	63.5 (19.1)	76.1 (17.8)
	FEV1/FVC	0.80 (0.09)	0.77 (0.12)
**Visit 2 (8 weeks)**	*Completed cases*	*n=15*	*n=11*
	FEV1	1.86 (0.54)	1.88 (0.59)
	FEV1 predicted	2.83 (0.60)	2.29 (0.51)
	FEV1 % predicted	66.3 (16.4)	81.8 (21.1)
	FVC	2.29 (0.75)	2.56 (0.75)
	FVC predicted	3.54 (0.69)	3.06 (0.69)
	FVC % predicted	65.3 (16.6)	84.2 (21.0)
	FEV1/FVC	0.82 (0.12)	0.73 (0.09)
**Visit 4 (16 weeks)**	*Completed cases*	*n=11*	*n=10*
	FEV1	1.84 (0.55)	1.94 (0.42)
	FEV1 predicted	2.68 (0.60)	2.38 (0.59)
	FEV1 % predicted	68.8 (16.4)	84.2 (18.5)
	FVC	2.37 (0.80)	2.63 (0.65)
	FVC predicted	3.49 (0.76)	3.18 (0.76)
	FVC % predicted	67.9 (15.4)	85.6 (24.9)
	FEV1/FVC	0.78 (0.05)	0.76 (0.06)

FEV1 - forced expiratory volume in 1 second; FVC - forced vital capacity.

Supplementary Table 3. The hospital anxiety and depression scale (**HADS**) measured at baseline, visit 1, 2, 3 and 4.

		**Wait list – mean (SD)**	**Fast track - mean (SD)**
**Baseline**	*Completed cases*	*n=25*	*n=22*
	Depression	7.4 (3.8)	7.3 (4.2)
	Anxiety	8.1 (3.9)	7.9 (4.5)
**Visit 1 (4 weeks)**	*Completed cases*	*n=20*	*n=21*
	Depression	7.4 (3.7)	6.6 (3.7)
	Anxiety	7.9 (4.2)	7.5 (4.1)
**Visit 2 (8 weeks)**	*Completed cases*	*n=23*	*n=20*
	Depression	7.3 (3.5)	7.2 (3.7)
	Anxiety	8.3 (3.1)	8.7 (3.8)
**Visit 3 (12 weeks)**	*Completed cases*	*n=19*	*n=18*
	Depression	7.4 (4.0)	7.4 (4.7)
	Anxiety	7.8 (3.6)	8.8 (6.0)
**Visit 4 (16 weeks)**	*Completed cases*	*n=18*	*n=15*
	Depression	7.2 (3.7)	6.1 (3.2)
	Anxiety	7.7 (3.5)	7.2 (3.6)

Supplementary Table 4. AKPS measured at baseline, visit 1, 2, 3 and 4.

		**Wait list**	**Fast track**
**Baseline**	*Completed cases*	*n=25*	*n=22*
	**Mean (SD)**	63.2 (9.0)	65.9 (10.1)
**Visit 1 (4 weeks)**	*Completed cases*	*n=21*	*n=21*
	**Mean (SD)**	65.7 (9.3)	66.7 (9.7)
**Visit 2 (8 weeks)**	*Completed cases*	*n=23*	*n=19*
	**Mean (SD)**	65.7 (10.8)	64.2 (9.0)
**Visit 3 (12 weeks)**	*Completed cases*	*n=17*	*n=17*
	**Mean (SD)**	66.5 (7.0)	62.9 (9.9)
**Visit 4 (16 weeks)**	*Completed cases*	*n=15*	*n=15*
	**Mean (SD)**	65.3 (7.4)	66.0 (8.3)

Supplementary Table 5. Incremental Shuttle Walk Test (ISWT) measured at baseline, visit 1, 2, 3 and 4.

		**Wait list – mean (SD)**	**Fast track - mean (SD)**
**Baseline**	*Completed cases*	*n=22*	*n=20*
	Total Number of shuttles	12.5 (12.2)	13.3 (11.3)
	Total Distance Walked (meters)	125 (122)	133(113)
**Visit 1 (4 weeks)**	*Completed cases*	*n=14*	*n=17*
	Total Number of shuttles	14.9 (12.7)	14.7 (11.7)
	Total Distance Walked (meters)	149 (127)	147 (117)
**Visit 2 (8 weeks)**	*Completed cases*	*n=16*	*n=13*
	Total Number of shuttles	16.4 (13.8)	12.1 (6.7)
	Total Distance Walked (meters)	164 (138)	121 (67)
**Visit 3 (12 weeks)**	*Completed cases*	*n=13*	*n=12*
	Total Number of shuttles	15.4 (12.6)	17.0 (15.7)
	Total Distance Walked (meters)	154 (126)	170 (157)
**Visit 4 (16 weeks)**	*Completed cases*	*n=12*	*n=10*
	Total Number of shuttles	17.8 (12.2)	14.8 (9.5)
	Total Distance Walked (meters)	178 (122)	148 (95)

Supplementary Table 6. Physical activity data derived from the Actigraph GT3XP-BT device at baseline and visits 1,2,3, and 4. Physical activity intensity was defined as per Freedson et al. (1998)[1].

		Wait list – mean (SD)	Fast track - mean (SD)
**Baseline**	*Completed cases*	*n=18*	*n=15*
	Number of valid days	4.9 (0.2)	5 (0)
	Wearing time (min)	5823 (1109)	6157 (666)
	Average steps per day	6569 (3665)	8432 (4672)
	Sedentary activity (min)	5103 (1080)	5314 (683)
	Sedentary activity (%)	87% (6%)	86% (6%)
	Light activity (min)	570 (196)	674(301)
	Light activity (%)	10% (3%)	11% (4%)
	Moderate/vigorous activity (min)	150 (124)	168 (127)
	Moderate/vigorous activity (%)	3% (4%)	3% (2%)
**Visit 1 (4 weeks)**	*Completed cases*	*n=10*	*n=10*
	Number of valid days	5 (0)	4.8 (0.6)
	Wearing time (min)	6087 (966)	5988 (1198)
	Average steps per day	8672 (2669)	8197 (3442)
	Sedentary activity (min)	5440 (899)	5334 (1068)
	Sedentary activity (%)	85% (5%)	87% (5%)
	Light activity (min)	740 (228)	681 (263)
	Light activity (%)	12% (4%)	11% (3%)
	Moderate/vigorous activity (min)	220 (105)	153 (110)
	Moderate/vigorous activity (%)	4% (2%)	2% (2%)
**Visit 2 (8 weeks)**	*Completed cases*	*n=8*	*n=10*
	Number of valid days	4.8 (0.5)	4.9 (0.3)
	Wearing time (min)	5918 (1205)	6053 (621)
	Average steps per day	8002 (4515)	8253 (5553)
	Sedentary activity (min)	5124 (1116)	5225 (478)
	Sedentary activity (%)	86% (6%)	87% (7%)
	Light activity (min)	613 (259)	661 (341)
	Light activity (%)	11% (4%)	11% (5%)
	Moderate/vigorous activity (min)	181 (162)	168 (117)
	Moderate/vigorous activity (%)	3% (3%)	3% (2%)
**Visit 3 (12 weeks)**	*Completed cases*	*n=7*	*n=11*

	Number of valid days	5 (0)	5 (0)
	Wearing time (min)	6217 (727)	6179 (633)
	Average steps per day	8061 (3448)	7570 (4815)
	Sedentary activity (min)	5368 (708)	5395 (654)
	Sedentary activity (%)	86% (4%)	87% (6%)
	Light activity (min)	637 (207)	649 (276)
	Light activity (%)	10% (3%)	10% (4%)
	Moderate/vigorous activity (min)	213 (101)	135 (133)
	Moderate/vigorous activity (%)	3% (1%)	2% (2%)
**Visit 4 (16 weeks)**	*Completed cases*	*n=6*	*n=7*
	Number of valid days	4.8 (0.4)	5 (0)
	Wearing time (min)	5892 (814)	6153 (418)
	Average steps per day	7980 (3584)	10096 (5070)
	Sedentary activity (min)	5049 (733)	5119 (421)
	Sedentary activity (%)	86% (6%)	83% (6%)
	Light activity (min)	629 (235)	866 (319)
	Light activity (%)	11% (4%)	14% (5%)
	Moderate/vigorous activity (min)	213 (124)	168 (89)
	Moderate/vigorous activity (%)	4% (2%)	3% (1%)

Supplementary Table 7. SGRQ-I measured at baseline, visit 1, 2, 3 and 4.

		**Wait list – mean (SD)**	**Fast track - mean (SD)**
**Baseline**	*Completed cases*	*n=25*	*n=22*
	Symptoms	76.1 (18.0)	72.9 (16.5)
	Activity	89.8 (14.8)	89.3 (13.2)
	Impacts	55.4 (18.3)	48.8 (24.0)
	Total score	68.6 (13.8)	64.3 (16.8)
**Visit 1 (4 weeks)**	*Completed cases*	*n=20*	*n=21*
	Symptoms	71.8 (19.1)	67.9 (21.2)
	Activity	86.3 (12.3)	86.2 (14.5)
	Impacts	55.5 (16.5)	46.9 (21.5)
	Total score	66.9 (13.5)	61.5 (16.8)
**Visit 2 (8 weeks)**	*Completed cases*	*n=23*	*n=20*
	Symptoms	77.9 (19.8)	70.2 (19.5)
	Activity	92.0 (10.5)	88.7 (14.1)
	Impacts	54.8 (15.4)	51.9 (22.0)
	Total score	69.2 (11.9)	65.3 (15.8)
**Visit 3 (12 weeks)**	*Completed cases*	*n=19*	*n=17*
	Symptoms	69.9 (21.7)	77.1 (15.6)
	Activity	89.5 (14.2)	90.9 (11.1)
	Impacts	45.4 (19.7)	50.6 (23.2)
	Total score	61.9 (15.1)	66.4 (15.3)
**Visit 4 (16 weeks)**	*Completed cases*	*n=18*	*n=16*
	Symptoms	74.5 (18.4)	72.6 (16.4)
	Activity	89.1 (9.7)	88.4 (14.6)
	Impacts	54.8 (21.9)	49.8 (20.3)
	Total score	67.8 (15.8)	64.6 (14.3)

Supplementary Table 8a. Adverse event data for both groups.

		Wait list – n (%)	Fast track - n (%)
Total number of Adverse Events		*n=28*	*n=29*
**Intensity**	Mild	15 (53.6%)	12 (41.4%)
	Moderate	11 (39.3%)	13 (44.8%)
	Severe	2 (7.1%)	4 (13.8%)
**Seriousness**	Serious	2 (7.1%)	3 (10.3%)
	Non-serious	26 (92.9%)	26 (89.7%)
**Causality**	Suspected	1 (3.6%)	0 (0%)
	Non-suspected	27 (96.4%)	29 (100%)
**Action taken with study**	Intervention not changed	9 (32.1%)	4 (13.8%)
	Intervention withdrawn	1 (3.6%)	0 (0%)
	Not applicable	18 (64.3%)	24 (82.8%)
	Unknown	0 (0%)	1 (3.4%)
**Outcome**	Fatal	1 (3.6%)	3 (10.3%)
	Not recovered/not resolved	3 (10.7%)	7 (24.1%)
	Recovered/Resolved	16 (57.1%)	10 (34.5%)
	Recovered/Resolved with sequelae	0 (0%)	1 (3.4%)
	Unknown	2 (7.1%)	3 (10.3%)

Supplementary Table 8b. Adverse event data for both groups

**Adverse Event Description**	**Number (%) of patients reporting adverse events**	
	**Wait-List (n=25)**	**Fast-Track (n=22)**
**All adverse events**	**15 (60%)**	**15 (68)**
**Respiratory**	**7 (28%)**	**8 (36)**
- Increased Breathlessness	2 (8%)	4 (18)
- Severe Cough	3 (12%)	0 (0)
- 'Chest Infection'	4 (16%)	4 (18)
**Non-Respiratory***	**12 (48%)**	**10 (50)**
**Serious Adverse Events**	**2 (8%)**	**3 (14)**
**Respiratory**	**1 (4%)**	**3 (14)**
- Increased breathlessness	1 (4%)	3 (14)
**Non-respiratory***	**1 (4%)**	**0 (0)**
**Deaths**	**1 (4%)**	**2 (9)**
**Respiratory**	**0 (0%)**	**2 (9)#**
**Non-respiratory***	**1 (4%)**	**0 (0)**

Table 7b. Number of participants reporting respiratory and non-respiratory adverse events by group allocation. *a range of non-respiratory adverse events were reported by participants with low frequency including: acute coronary syndrome (fatal), ankle/joint swelling, dental abscess, diarrhoea, fall, fatigue/lethargy, gastroesophageal reflux, hernia, hypertension, iron deficiency, muscle spasms, urinary tract infection, valvular heart disease and weight loss. # one death was attributed to increased shortness of breath due to heart failure and IPF.

Supplementary Table 9. EQ-5D-5L measured at baseline, visit 1, 2, 3 and 4.

		**Wait list – mean (SD)**	**Fast track - mean (SD)**
**Baseline**	*Completed cases*	*n=25*	*n=22*
	Utility score	0.59 (0.19)	0.53 (0.21)
	EQ-VAS score	56.1 (18.0)	60.2 (20.2)
**Visit 1 (4 weeks)**	*Completed cases*	*n=20*	*n=21*
	Utility score	0.57 (0.28)	0.55 (0.23)
	EQ-VAS score	55.3 (19.5)	60.2 (18.9)
**Visit 2 (8 weeks)**	*Completed cases*	*n=23*	*n=20*
	Utility score	0.53 (0.16)	0.56 (0.17)
	EQ-VAS score	52.3 (19.1)	56.3 (16.0)
**Visit 3 (12 weeks)**	*Completed cases*	*n=19*	*n=18*
	Utility score	0.61 (0.17)	0.54 (0.24)
	EQ-VAS score	61.3 (17.1)	53.4 (18.1)
**Visit 4 (16 weeks)**	*Completed cases*	*n=18*	*n=16*
	Utility score	0.57 (0.16)	0.58 (0.20)
	EQ-VAS score	58.3 (15.0)	61.8 (18.6)

Supplementary Table 10. Health Care Utilisation measured at **Visit 1**

				**Wait list – n (%)**	**Fast track - n (%)**
Total number of recorded				*n=21*	*n=21*
**General practitioner**	Yes			7 (33.3%)	6 (28.6%)
		If yes	1 utilisation	6	6
			3 utilisation	1	0
	No			14 (66.7%)	15 (71.4%)
**Practice nurse**	Yes			6 (28.6%)	3 (14.3%)
		If yes	1 utilisation	4	3
			2 utilisation	1	0
			missing	1	0
	No			15 (71.4%)	18 (85.7%)
**Occupational health**	Yes			0 (0%)	2 (9.5%)
		If yes	1 utilisation	0	2
	No			21 (100%)	19 (90.5%)
**Other health**	Yes			4 (19.0%)	3 (14.3%)
		If yes	1 utilisation	4	3
	No			17 (81.0%)	18 (85.7%)
**Accident and emergency**	Yes			0 (0%)	0 (0%)
	No			21 (100%)	21 (100%)
**NHS walking in centre**	Yes			0 (0%)	0 (0%)
	No			21 (100%)	21 (100%)
**NHS urgent care centre**	Yes			0 (0%)	0 (0%)
	No			21 (100%)	21 (100%)
**Hospital outpatient appointment**	Yes			7 (33.3%)	6 (28.6%)
		If yes	1 utilisation	5	6
			2 utilisation	1	0
			missing	1	0
	No			14 (66.7%)	15 (71.4%)
**Has patient been admitted**	Yes			0 (0%)	0 (0%)
	No			21 (100%)	21 (100%)
**Has patient been admitted nursing home**	Yes			0 (0%)	0 (0%)
	No			21 (100%)	21 (100%)

Supplementary Table 11. Health Care Utilisation measured at **Visit 2**

				**Wait list – n (%)**	**Fast track - n (%)**
Total number of recorded				*n=23*	*n=20*
**General practitioner**	Yes			9 (39.1%)	8 (40.0%)
		If yes	1 utilisation	7	5
			2 utilisation	1	3
			3 utilisation	1	0
	No			14 (60.9%)	12 (60%)
**Practice nurse**	Yes			5 (21.7%)	3 (15.0%)
		If yes	1 utilisation	5	2
			missing	0	1
	No			18 (78.3%)	17 (85.0%)
**Occupational health**	Yes			1 (4.3%)	1 (5.0%)
		If yes	1 utilisation	1	1
	No			22 (95.7%)	19 (95.0%)
**Other health**	Yes			2 (8.7%)	3 (15.0%)
		If yes	1 utilisation	2	3
	No			21 (91.3%)	17 (85.0%)
**Accident and emergency**	Yes			1 (4.3%)	0 (0%)
		If yes	missing	1	0
	No			22 (95.7%)	20 (100%)
**NHS walking in centre**	Yes			1 (4.3%)	0 (0%)
		If yes	1 utilisation	1	0
	No			22 (95.7%)	20 (100%)
**NHS urgent care centre**	Yes			0 (0%)	0 (0%)
	No			23 (100%)	20 (100%)
**Hospital outpatient appointment**	Yes			7 (30.4%)	8 (40%)
		If yes	1 utilisation	6	6
			2 utilisation	0	1
			missing	1	1
	No			16 (69.6%)	11 (55%)
	Missing			0 (0%)	1 (5%)
**Has patient been admitted**	Yes			0 (0%)	0 (0%)
	No			23 (100%)	19 (95%)
	Missing			0 (0%)	1 (5%)
**Has patient been admitted nursing home**	Yes			0 (0%)	0 (0%)
	No			23 (100%)	19 (95%)
	Missing			0 (0%)	1 (5%)

Supplementary Table 12. Health Care Utilisation measured at **Visit 3**

			Wait list – n (%)	Fast track - n (%)
Total number of recorded			*n=20*	*n=19*
**General practitioner**	Yes		9 (45%)	6 (31.6%)
	If yes	1 utilisation	8	4
		2 utilisation	0	1
		4 utilisation	1	1
	No		11 (55%)	13 (68.4%)
**Practice nurse**	Yes		6 (30%)	4 (21.1%)
	If yes	1 utilisation	5	4
		2 utilisation	1	0
	No		14 (70%)	15 (78.9%)
**Occupational health**	Yes		1 (5%)	1 (5.3%)
	If yes	1 utilisation	1	1
	No		19 (95%)	18 (94.7%)
**Other health**	Yes		2 (10%)	4 (21.1%)
	If yes	1 utilisation	2	3
		2 utilisation	0	1
	No		18 (90%)	15 (78.9%)
**Accident and emergency**	Yes		0 (0%)	1 (5.3%)
	If yes	1 utilisation	0	1
	No		20 (100%)	18 (94.7%)
**NHS walking in centre**	Yes		0 (0%)	0 (0%)
	No		20 (100%)	18 (94.7%)
	Missing		0 (0%)	1 (5.3%)
**NHS urgent care centre**	Yes		0 (0%)	0 (0%)
	No		20 (100%)	19 (100%)
**Hospital outpatient appointment**	Yes		4 (20%)	4 (21.1%)
	If yes	1 utilisation	3	2
		2 utilisation	0	2
		missing	1	0
	No		16 (80%)	15 (78.9%)
**Has patient been admitted**	Yes		0 (0%)	2 (10.5%)
	If yes, no. of days	1 day	0	1
		2 days	0	1
	No		20 (100%)	17 (89.5%)
**Has patient been admitted nursing home**	Yes		0 (0%)	0 (0%)
	No		20 (100%)	19 (100%)

Supplementary Table 13. Health Care Utilisation measured at **Visit 4**

				Wait list – n (%)	Fast track - n (%)
Total number of recorded				*n=19*	*n=16*
**General practitioner**	Yes			5 (26.3%)	6 (37.5%)
		If yes	1 utilisation	4	3
			2 utilisation	1	3
	No			14 (73.7%)	10 (62.5%)
**Practice nurse**	Yes			5 (26.3%)	3 (18.8%)
		If yes	1 utilisation	4	3
			6 utilisation	1	0
	No			14 (73.7%)	13 (81.3%)
**Occupational health**	Yes			0 (0%)	0 (0%)
	No			19 (100%)	16 (100%)
**Other health**	Yes			1 (5.3%)	1 (6.3%)
		If yes	1 utilisation	0	1
			4 utilisation	1	0
	No			18 (94.7%)	15 (93.8%)
**Accident and emergency**	Yes			1 (5.3%)	1 (6.3%)
		If yes	1 utilisation	1	1
	No			18 (94.7%)	15 (93.8%)
**NHS walking in centre**	Yes			0 (0%)	0 (0%)
	No			19 (100%)	16 (100%)
**NHS urgent care centre**	Yes			0 (0%)	0 (0%)
	No			19 (100%)	16 (100%)
**Hospital outpatient appointment**	Yes			3 (15.8%)	3 (18.8%)
		If yes	1 utilisation	3	2
			2 utilisation	0	1
	No			16 (84.2%)	13 (81.3%)
**Has patient been admitted**	Yes			0 (0%)	0 (0%)
	No			19 (100%)	16 (100%)
**Has patient been admitted nursing home**	Yes			0 (0%)	0 (0%)
	No			19 (100%)	15 (93.8%)
	Missing			0 (0%)	1 (6.3%)

		Wait list – n (%)					Fast track - n (%)				
	Completed cases	*n=25*	*n=22*	*n=23*	*n=19*	*n=18*	*n=22*	*n=21*	*n=20*	*n=18*	*n=16*
		Baseline	*V1*	*V2*	*V3*	*V4*	*Baseline*	*V1*	*V2*	*V3*	*V4*
**Having a say**	Never	0 (0%)	0 (0%)	0 (0%)	0 (0%)	0 (0%)	0 (0%)	0 (0%)	0 (0%)	0 (0%)	0 (0%)
	Only a little of the time	0 (0%)	1 (5%)	1 (4.3%)	0 (0%)	0 (0%)	0 (0%)	1 (4.8%)	1 (5%)	0 (0%)	1 (6.3%)
	Some of the time	4 (16.0%)	0 (0%)	0 (0%)	2 (10.5%)	2 (11.1%)	1 (4.5%)	0 (0%)	2 (10%)	1 (5.6%)	0 (0%)
	Most of the time	21 (84.0%)	19 (95%)	22 (95.7%)	17 (89.5%)	16 (88.9%)	21 (95.5%)	20 (95.2%)	17 (85%)	17 (94.4%)	15 (93.8%)
**Being with people who care about you**	Never be able to	0 (0%)	0 (0%)	0 (0%)	0 (0%)	0 (0%)	0 (0%)	0 (0%)	0 (0%)	0 (0%)	0 (0%)
	Only a little of the time	0 (0%)	1 (5%)	3 (13.0%)	1 (5.3%)	2 (11.1%)	1 (4.5%)	2 (9.5%)	1 (5%)	3 (16.7%)	1 (6.3%)
	Some of the time	4 (16.0%)	4 (20%)	3 (13.0%)	3 (15.8%)	4 (22.2%)	3 (13.6%)	4 (19.0%)	6 (30%)	2 (11.1%)	3 (18.8%)
	Most of the time	21 (84.0%)	15 (75%)	17 (73.0%)	15 (78.9%)	12 (66.7%)	18 (81.8%)	15 (71.4%)	13 (65%)	13 (72.2%)	12 (75.0%)
**Physical suffering**	Always	1 (4.0%)	3 (15%)	2 (8.7%)	3 (15.8%)	*3 (16.7%)*	5 (22.7%)	1 (4.8%)	1 (5%)	4 (22.2%)	*3 (18.8%)*
	Often	10 (40.0%)	6 (30%)	7 (30.4%)	7 (36.8%)	6 (33.3%)	7 (31.8%)	6 (28.6%)	6 (30%)	4 (22.2%)	2 (12.5%)
	Sometimes	10 (40.0%)	7 (35%)	10 (43.5%)	5 (26.3%)	6 (33.3%)	7 (31.8%)	7 (33.3%)	10 (50%)	8 (44.4%)	9 (56.3%)
	Rarely	4 (16.0%)	4 (20%)	4 (17.4%)	4 (21.1%)	3 (16.7%)	3 (13.6%)	7 (33.3%)	3 (15%)	2 (11.1%)	2 (12.5%)
**Emotional suffering**	Always	1 (4.0%)	0 (0%)	0 (0%)	1 (5.3%)	0 (0%)	2 (9.1%)	2 (9.5%)	0 (0%)	1 (5.6%)	0 (0%)
	Often	4 (16.0%)	4 (20%)	7 (30.4%)	2 (10.5%)	4 (22.2%)	6 (27.3%)	5 (23.8%)	3 (15%)	6 (33.3%)	2 (12.5%)
	Sometimes	13 (52.0%)	5 (25%)	11 (47.8%)	11 (57.9%)	6 (33.3%)	6 (27.3%)	6 (28.6%)	11 (55%)	5 (27.8%)	7 (43.8%)
	Rarely	7 (28.0%)	11 (55%)	5 (21.7%)	5 (26.3%)	8 (44.4%)	8 (36.4%)	8 (38.1%)	6 (30%)	6 (33.3%)	7 (43.8%)
**Dignity**	Never	0 (0%)	0 (0%)	0 (0%)	0 (0%)	*0 (0%)*	0 (0%)	0 (0%)	1 (5%)	0 (0%)	*0 (0%)*
	Only a little of the time	0 (0%)	0 (0%)	0 (0%)	0 (0%)	0 (0%)	1 (4.5%)	0 (0%)	0 (0%)	0 (0%)	1 (6.3%)
	Some of the time	2 (8.0%)	1 (5%)	3 (13.0%)	1 (5.3%)	1 (5.6%)	2 (9.1%)	3 (14.3%)	3 (15%)	2 (11.1%)	1 (6.3%)
	Most of the time	23 (92.0%)	19 (95%)	20 (87.0%)	18 (94.7%)	17 (94.4%)	19 (86.4%)	18 (85.7%)	16 (70%)	16 (88.9%)	14 (87.5%)
**Being supported**	Never	0 (0%)	0 (0%)	1 (4.3%)	0 (0%)	0 (0%)	0 (0%)	0 (0%)	0 (0%)	0 (0%)	0 (0%)
	Only a little of the time	2 (8.0%)	3 (15%)	2 (8.7%)	1 (5.3%)	2 (11.1%)	1 (4.5%)	2 (9.5%)	1 (5%)	3 (16.7%)	1 (6.3%)
	Some of the time	3 (12.0%)	3 (15%)	4 (17.4%)	1 (5.3%)	0 (0%)	4 (18.2%)	4 (19.0%)	5 (25%)	2 (11.1%)	3 (18.8%)
	Most of the time	20 (80.0%)	14 (70%)	16 (69.6%)	17 (89.5%)	16 (88.9%)	17 (77.3%)	15 (71.4%)	14 (70%)	13 (72.2%)	12 (75.0%)
**Being prepared**	Not any	1 (4%)	1 (5%)	1 (4.3%)	1 (5.3%)	1 (5.6%)	1 (4.5%)	2 (9.5%)	0 (0%)	1 (5.6%)	0 (0%)
	Few	5 (20.0%)	2 (10%)	2 (8.7%)	1 (5.3%)	1 (5.6%)	2 (9.1%)	1 (4.8%)	1 (5%)	0 (0%)	1 (6.3%)
	Some	4 (16.0%)	4 (20%)	8 (17.4%)	4 (21.1%)	4 (5.6%)	6 (27.3%)	4 (19.0%)	5 (25%)	2 (11.1%)	1 (6.3%)
	Most	15 (60.0%)	13 (65%)	12 (69.6%)	13 (68.4%)	12 (66.7%)	13 (59.1%)	14 (66.7%)	14 (70%)	15 (83.3%)	14 (87.5%)

Supplementary Table 14. ICECAP-SCM data for fast-track and wait-list groups at all study visit. V1: Visit 1; V2: Visit 2; V3: Visit 3; V4: Visit 4.

QUANT Data	QUANT findings	QUAL themes	QUAL quotes	Synthesis
Recruitment: **eligibility:consent** ratio<br><br><br>**Recruitment** and **retention** rate	64 eligible and 50 consented.<br><br><br><br>Recruitment rate by site was uneven- range 2.2 to 0.12 per month	Some eligible patients declined because they felt they were beyond help. Family members could be a barrier to recruitment.<br><br><br>Barriers to recruitment<br>The number of appointments put patients off. This issue affected Scarborough more than Hull or York.	"There was someone else who was interested but her family didn't want her to have the hassle, […] she came on the phone it was, my son feels that I've got enough on" Rn13<br><br>"Some were initially quite keen and then when they read the information sheet some declined because of the number of visits […] some people lived just that bit too far out for them to come so frequently". Phy16	Recruitment to target is possible, but selection of sites important to ensure they have patients with eligible profile.<br><br>Refine eligibility process to identify those most likely to benefit. Is it reasonable for those who are seriously ill to be included? Reduce number of measures. Is attendance at clinic needed for all appointments?
To assess the **acceptability and fidelity** of the **intervention** by measuring **adherence** in delivery and uptake. This includes documenting aspects of breathlessness interventions used in the **control arm** provided ad hoc e.g. breathing techniques, use of the hand-held fan etc	Quantitative process data indicates therapists delivered all components, so intervention **delivered with fidelity.**	**Staff:** adapted the intervention depending on patient ability, and selected components based on their needs and preferences and finding what was useful for them<br><br>**Patient adherence to intervention elements**<br>Those who used **rectangular breathing** found it helped them feel less panicked.<br>Not everyone had a CD player, and felt similar/better **relaxation tracks** are available.<br>Fewer patients had practiced the **positions** suggested, but those that did found they helped.<br>Most found the **fan** useful, but for some it lacks credibility so they used it in private.<br>Not all participants completed the **exercises.** People who could, considered this element positive and useful.<br><br>Staff questioned the appropriateness of the intervention for those in the later stages of their illness.	"I saw it as like a bespoke intervention for each patient, so each patient was seen as an individual. They were kind of taught the same methods, but obviously it was dependent on the patient's ability to understand that and what they were able to do" Rn12<br><br>"It's the breathing, yeah, I would say [most useful]" 1P<br><br><br>[demonstrates open chest position] That's the one that helps." 1P<br><br>"You don't want to be bringing a fan out, it's like personal pride type of thing, you know, but I've got one in the car" 7P<br>Yes, I, I do use it [mobility component of the intervention] every day and, and at varying levels shall we say?" 7P<br><br>"if people are more poorly, they're probably, not, not engaged as well as other people who were maybe a bit earlier on in their journey, so it did depend on the health of a patient basically." 15Phy	All components delivered by therapists as required by the manual, but delivery tailored to the needs of the individual.<br><br>Patients engagement influenced by memory problems, level of physical functioning, and face validity of the intervention.<br><br>Patients selected those which they felt were most helpful to them. So, does intervention content need to be monitored?<br><br>Eligibility criteria need reviewing as some patients were too ill to engage with some aspects of intervention.
Data quality: To assess the amount and pattern of missing data for study measures. Data variability	100% for CRQ, HADS, NRS baseline and 87% for 4 week follow up	Patients struggled to attend appointments, particularly in winter months, resulting in missing physiological data. The burden of repeat visits was remarked on by patients to staff.	"I certainly didn't hear anyone talk about the, the nitty gritty, just the visits, just the burden of the visits." Rn13<br><br>"You've got a twenty four page questionnaire, they were okay but they were much the same weren't they?" 6C	As data collection was clinic based, poor weather and declining health in the patient affect data quality.

across outcome measures. These findings will inform the choice of primary and secondary outcomes for a definitive trial.	CRQ to be Primary outcome<br><br>At 16 weeks n=18 intervention and n=16 controls have data reported from CRQ.<br><br>Spirometry data at 16 weeks lower n=11 intervention and n=10 controls	Patients were happy with questionnaires but viewed them as repetitive.<br><br>Practitioners reported patients found questionnaires lengthy and confusing, especially with their repetitive nature.<br><br>Spirometry and shuttle walk test data missing as some patients declined in health during intervention and start of COVID.	"He'd like say oh well, you know, I'm," I feel happy" and then another one [question] would say "are you sad?" I think some got a bit flummoxed by all these questions. I think it was too much, yeah, it's just mind boggling." Rn11<br><br>"the shuttle walk and the spirometry, some of them had been missed due to the fact that they're [patient] just not well enough" Rn11	The number of physiological measures need to be reduced to increase retention and data quality.<br><br>Repetition in questionnaires needs to be removed (reduce number of measures).
4. Outcome: To assess the best primary outcome and agree other study measures for the definitive trial	Primary outcome – CRQ – mastery domain<br><br>NRS breathlessness – best (not good ES), worst, caused stress, how well coped.<br><br>Patients on intervention scored better	Some patients not like shuttle walk test, but staff liked the objective nature of the test.<br><br>Patients generally liked questionnaires but found them repetitive. Practitioners some concerns about patient comprehension and preferred objective assessments.	Well I wouldn't say it [walk] was really difficult but it's very, very tiring." 3P<br><br>"The only worse bits for me is when they, was the lung function tests because it really sets me off" 5P<br><br>"Oh they're (questionnaires) quite good, a lot, yeah, I thought they were [relevant], yeah." 4P	Divergence in views of patients and staff. Patients like questionnaires – probably because physiological measures are tiring. Staff like physiological measures as questionnaires rely on subjective/self report.

Supplementary Table 15. Mixed methods analysis

References

1. Freedson PS, Melanson E, Sirard J. Calibration of the Computer Science and Applications, Inc. accelerometer. Med Sci Sports Exerc. 1998 May;30(5):777-81.
